# Supplementary material for: Ballistic tracks in graphene nanoribbons
Source: Nat Commun. 2018 Oct 24;9:4426. doi: 10.1038/s41467-018-06940-5 (PMC6200825; doi:10.1038/s41467-018-06940-5)
Supplement: Supplementary file 1 — Supplementary Information [file 41467_2018_6940_MOESM1_ESM.pdf]

Ballistic tracks in graphene nanoribbons

Johannes Aprojanz[1], Stephen R. Power[2,3,4], Pantelis Bampoulis[5,6], Stephan Roche[2,7], Antti-Pekka Jauho[8], Harold J.W. Zandvliet[5], Alexei A. Zakharov[9] & Christoph Tegenkamp[1,6]

[1]*Institut für Physik, Technische Universität Chemnitz, 09126 Chemnitz, Germany*

[2]*Catalan Institute of Nanoscience and Nanotechnology (ICN2), CSIC and The Barcelona Institute of Science and Technology, Campus UAB, Bellaterra, 08193 Barcelona (Cerdanyola del Vallès), Spain*

[3]*Universitat Autònoma de Barcelona, 08193 Bellaterra (Cerdanyola del Vallès), Spain*

[4]*School of Physics, Trinity College Dublin, Dublin 2, Ireland*

[5]*Physics of Interfaces and Nanomaterials, MESA+ Institute for Nanotechnology, University of Twente, 7522 NH Enschede, The Netherlands*

[6]*Institut für Festkörperphysik, Leibniz Universität Hannover, 30167 Hannover, Germany*

[7]*ICREA, Institució Catalana de Recerca i Estudis Avançats, 08070 Barcelona, Spain*

[8]*Center for Nanostructured Graphene (CNG), DTU Nanotech, Technical University of Denmark, DK-2800, Kongens Lyngby, Denmark*

[9]*MAX IV Laboratory and Lund University, 221 00 Lund, Sweden*

The supplementary information contains details about: 1. Local transport measurements 2. AFM measurements 3. Tight-binding calculations

Supplementary Note 1, Local transport measurements

The growth of graphene nanoribbons at the sidewalls of SiC mesa structures is unavoidably accompanied by growth of graphene islands on SiC(0001) terraces. However, these islands are not percolated, thus do not influence our transport measurements. Our refined recipes enable us to suppress the growth of these parasitic structures down to the minimum. Nonetheless, the observation of small amounts of non-percolated graphene patches on the flat SiC(0001) terraces ensures that continuous ribbons are grown on the facets (cf. SEM and STM images in Supplementary Figure 1).

In our previous studies on ballistic sidewall ribbons, the probe spacing was always larger than

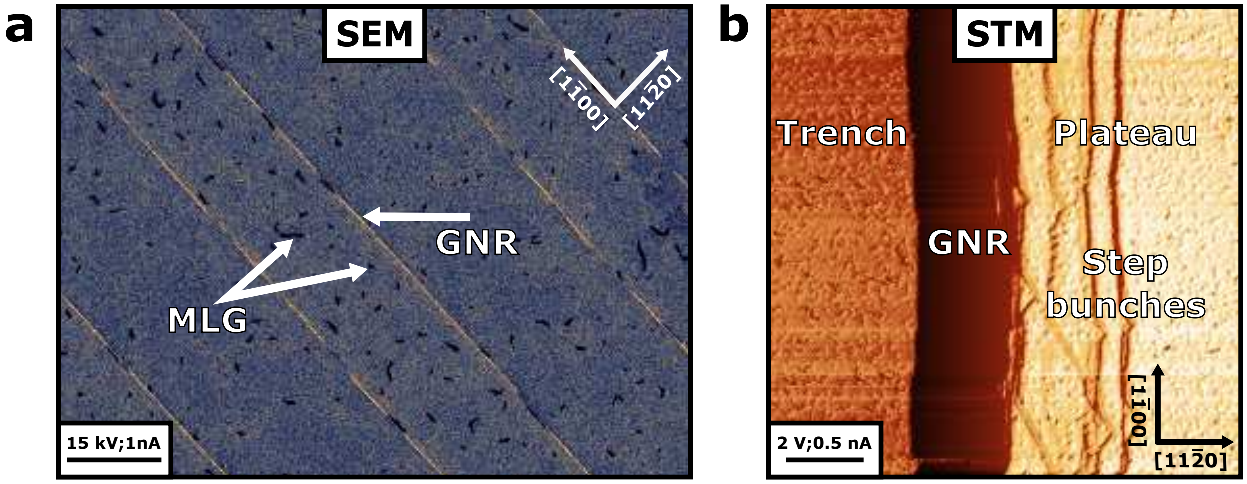

Supplementary Figure 1 | STM and SEM image of ballistic ribbons. **a** Large area SEM image showing the parasitic growth of non-percolated monolayer graphene (MLG) islands on the SiC(0001) terrace sites. **b** 2D STM topography of the inset of Fig. 1b depicts an entirely overgrown sidewall GNR besides some signatures of step-bunching located on the top part of the mesa structure. Scale bar corresponds to a length of 5 μm and 40 nm, respectively.

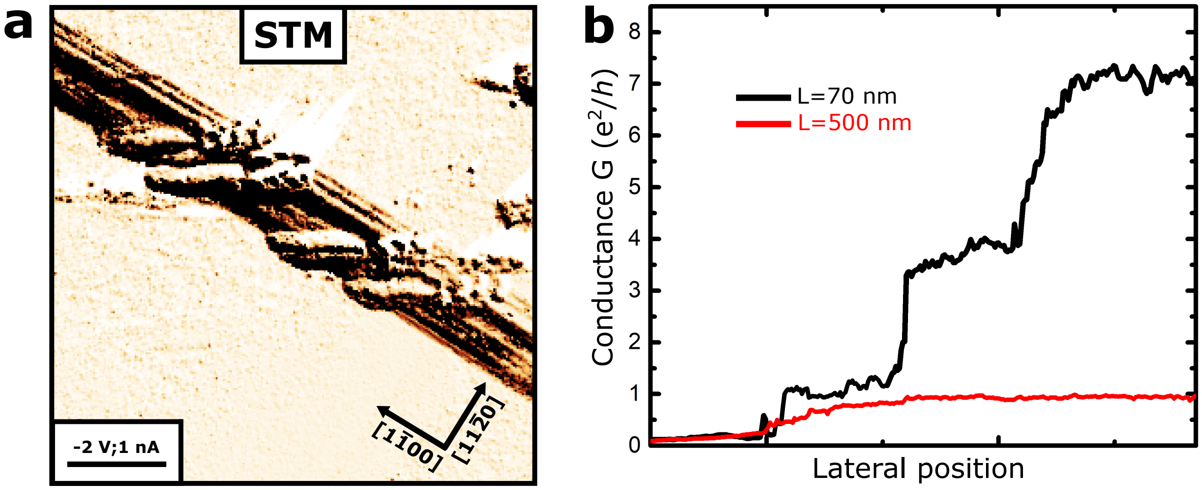

Supplementary Figure 2 | Spatially resolved 2pp transport measurements. a STM current image of GNR after transport measurements. Scale bar, 100 nm. The distance in between the residuals allows to determine the correct probe spacing. **b** Conductance G measured for two different distances L on the same ribbon at room temperature. A blunt tip was contacting the entire ribbon while a sharp tip was gradually moved across the ribbon starting from the lower edge (U=200 mV).

100 nm [1,2]. Generally, the finite apexes of the tips make it difficult to measure the probe spacing directly by SEM at smaller scales. To overcome this problem, the tips were pressed strongly against the ribbon when the transport study was completed, allowing to image the tip residuals by STM and to determine the probe spacing accurately (cf. Supplementary Figure 2a). Supplementary Figure 2b depicts spatially resolved 2pp transport measurements performed with two different fixed probe spacings. In accordance with previous results solely the spin-polarized temperature independent h/e^2 channel was measured for distances larger than 100 nm. The occurence of the fourfold degenarated channels at a probe spacing L = 70 nm is in full agreement with the limiting mean free path of a confined graphene sheet $l \sim 3\pi W/4 = 95\,\mathrm{nm}$ assuming a width of $W = 40\,\mathrm{nm}$

[4]. The systematic characterization of many ribbons shows the bonding of the lower edge and the formation of a sufficiently large suspended area to be the most crucial requirements for achieving quantised transport, whereas variations of the width (20-50 nm) or the doping of the 6H-SiC(0001) substrates do not affect this universal behavior.

Supplementary Note 2, AFM measurements

The topography and current images presented in Fig. 3a were recorded with a Pt tip, with a nominal radius of curvature of approximately 10 nm. Tip convolution effects induced by the finite tip size might influence the topographic image. In order to reduce convolution effects and avoid missing any topographic features present on the GNR walls, we have investigated the topography of the ribbons by the use of an ultra-sharp (radius of curvature 1-5 nm) diamond tip (AD-E-0.5-SS; Adama Innovations Ltd.). A representative topographic image is shown in Supplementary Figure 3a, showing a morphologically flat ribbon. This is further enhanced in the 3D image of Supplementary Figure 3b. Furthermore, by recording the torsional bending of the cantilever during contact mode scanning we can simultaneously obtain, along with the topographic image, a lateral force image. The lateral force image, see Supplementary Figure 3c, is the signal corresponding to the torsional bending of the cantilever. It gives information regarding topographic features, e.g., on a step edge the cantilever will bend, and material contrast. This is because when the tip moves horizontally across the surface, tip-surface forces result in a lateral deflection of the cantilever. The magnitude of the lateral forces depends on the friction coefficient of the surface, the topography and the mechanical properties of the cantilever. Lateral Force Microscopy is in principle more

sensitive than contact mode topography, providing information of the surface morphology with high spatial resolution, it can even resolve the lattice periodicity of the surface [3]. In order to avoid irreversible damage of the surface we have used normal forces in the order of a few nN. In

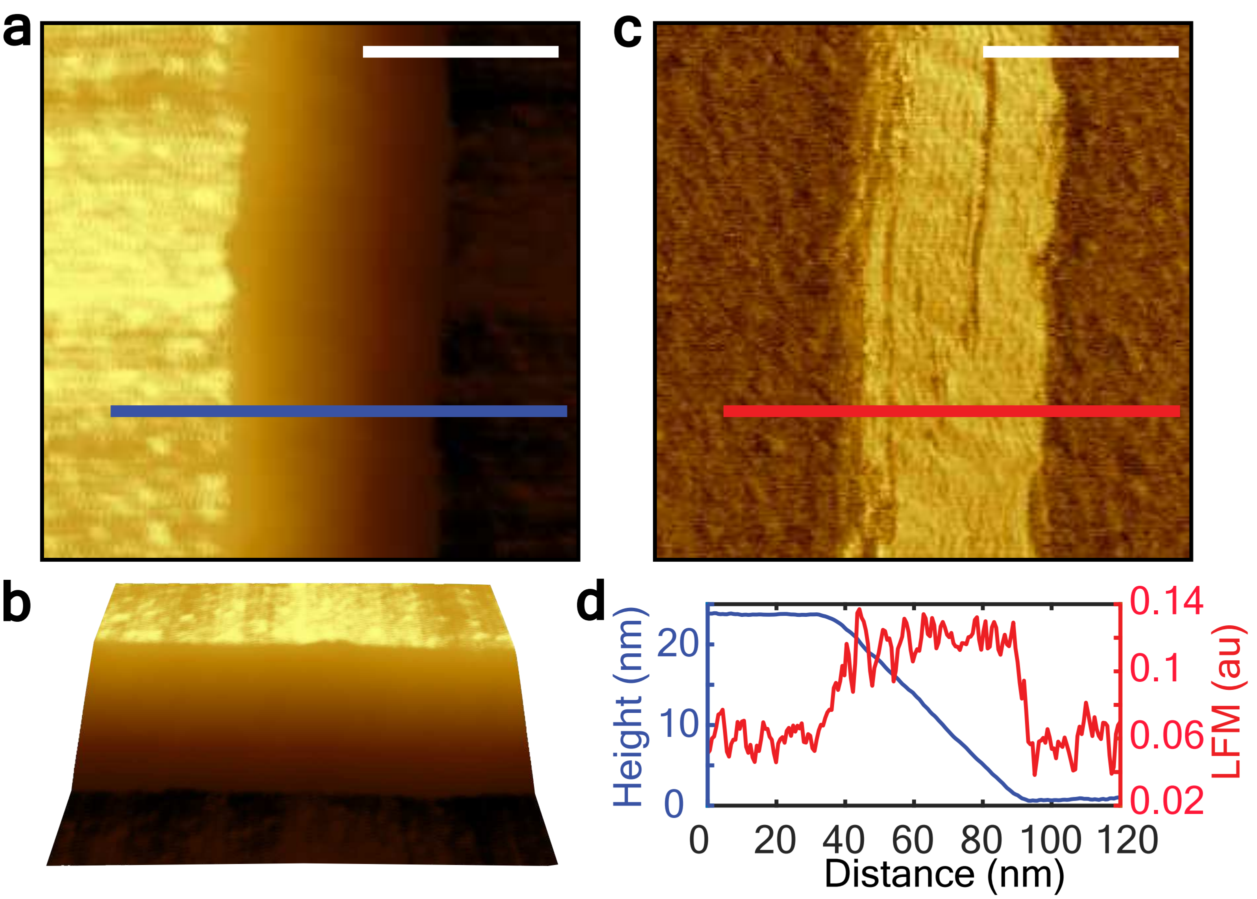

Supplementary Figure 3 | AFM measurements with diamond tips. a Topographic AFM image recorded with a high resolution diamond tip. The ribbon is morphologically flat. This is also demonstrated in the 3D image of panel **b**. **c** The simultaneously recorded with **a**, lateral force image, showing two clearly visible contrasts, corresponding to the GNR and the buffer layer. Scale bar in **a** and **c**, 50 nm. **d** Line profiles recorded across the ribbon of panel **a** and **c**, at the location indicated with the white line.

Supplementary Figure 3c,d the lateral force image and the corresponding cross sections clearly show that the material on the facet (ribbon) is different compared to its surroundings, since there is a clear color contrast. In addition, the lateral force image is more detailed than the topographic image, showing a small roughness but overall displaying a morphologically flat ribbon.

Supplementary Note 3, Tight-binding calculations

Band structure and conductance plateaus. In Supplementary Figure 4, we show more details for the calculation discussed in the main text, and in particular, the robustness of the three-plateau feature over a wide range of energies. In panels (c, d) we repeat the band projection and conductance-versus-width calculations for additional energies on either side of the Dirac point. A similar qualitative picture to the main text case (black curve here), e.g. a clear three-plateau structure, is seen for most of these energies (Supplementary Figure 4d). We can relate this to a similar distribution of states for these energies in Supplementary Figure 4c – the first four columns represent doubly-degenerate bulk-type states, and for the majority of energies we find two on each side of the ribbon. For energies further from the Dirac point, but still within the W-shaped region (e.g. $0.025|t|$, dark blue), we note a change in behaviour due to a contribution from higher-order modes. This can be seen more clearly by comparing the central projection column for $0.025|t|$ (second row, dark blue) with that of $0.050|t|$ (top row, light blue), which from Supplementary Figure 4b clearly has contributions from two sub-bands (multiple sub-band contributions are also discussed in the following section). The clear three-plateau signature seen for the majority of energies here suggests that the experimental result should be robust once the Fermi energy is reasonably close to the Dirac point.

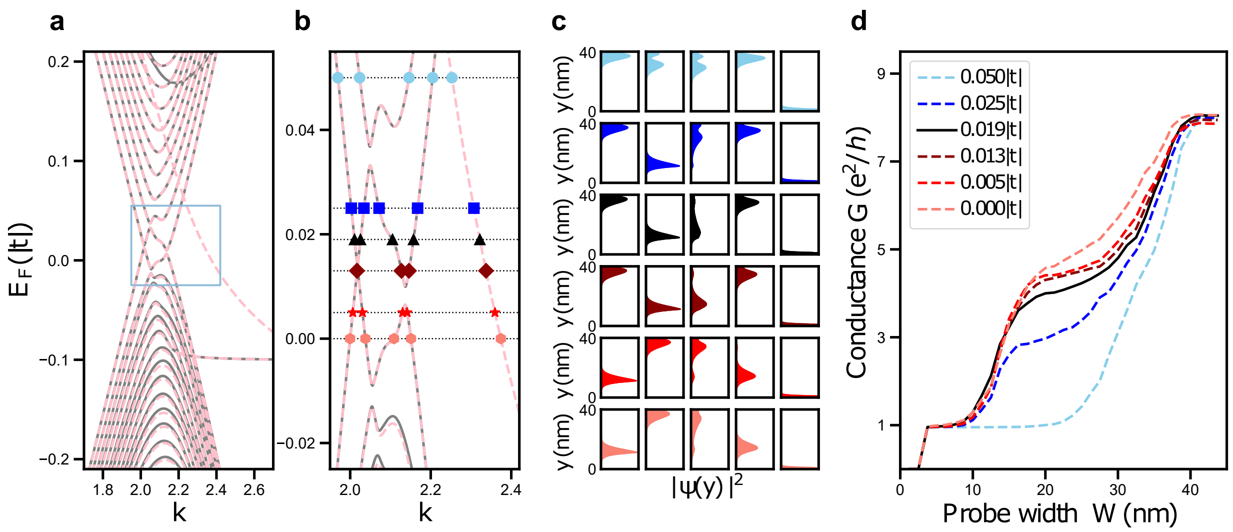

Supplementary Figure 4 | Tight-binding band-structures, band projections and conductance sweeps for the system considered in the main text. Panel **a** shows the band-structure for a wider range of energy and momenta values surrounding the K valley – a symmetric result is obtained for the K′ valley. Panel **b** shows a similar range to the main text, but highlights six specific Fermi energies, whose band projections and conductance sweeps (analogous to Fig. 4c,a in the main text) are shown in panels **c, d**. The majority of these energies show a qualitatively similar behaviour to that in the main text (solid, black curve here), the exceptions being energies further from the Dirac point (light and dark blue curves) where the bulk states cluster at one side of the ribbon due to contributions from higher sub-bands.

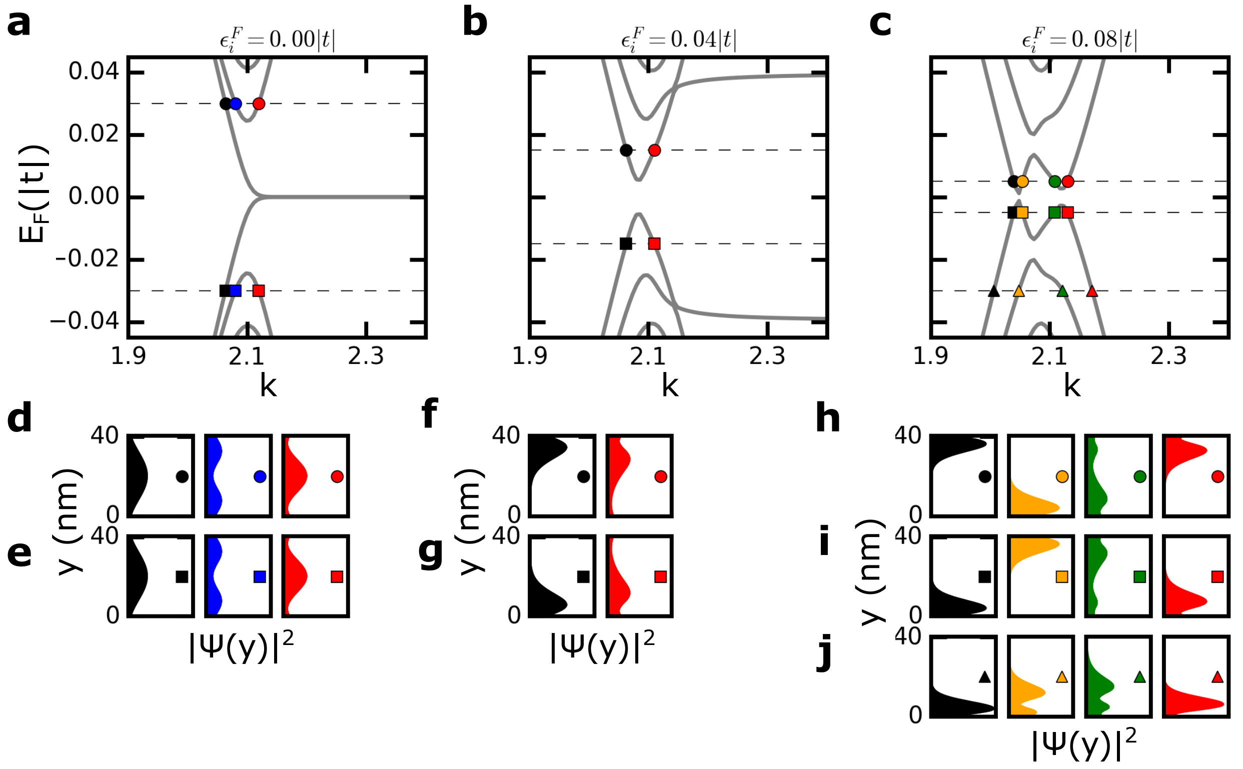

Supplementary Figure 5 | Tight-binding band-structures and band projections. a, b, c Electronic band-structures for simplified, non-magnetic ribbons, with the same width as those in the main manuscript, for increasing values of the transverse field parameter ϵ^{F}. Band crossings for Fermi energies in the conduction and valence bands, and associated crossing points, are shown by dashed lines and coloured symbols, respectively. Projections for the crossing points at symmetric conduction band **d, f, h** and valence band **e, g, i** Fermi energies around $E = 0$ show that these states are spread across the ribbon width when ϵ^{F} but are pushed towards the top (conduction) and bottom (valence) edges as this term increases. **j** Projections for a Fermi energy further from $E = 0$ show that all subbands are pushed towards the same edge, whereas additional channels due to band-bending at the lower energy **h, i** can reside on opposite edges.

Effect of transverse field term. In Supplementary Figure 5 we show in more detail the migration of bulk states towards the upper and lower edges of the ribbon as the transverse field term ϵ^{F} is increased. For clarity we set the other terms, corresponding to magnetic edge effects and local gap opening, to zero in these calculations. Moving from left to right (increasing ϵ^{F}), we note the following trends in the band structure: i) a splitting of the $E = 0$ edge states and their migration outside the studied energy window, ii) a closing of the gap between the lowest conduction and highest valence bulk bands, iii) a bending of these bands to form a W-shape once they begin to overlap, suggesting a hybridisation of states with valence and conduction band characteristics. Following the evolution of the black and red channels in panels (d, f, h) and (e, g, i) we see that those associated with conduction (valence) bands near the Fermi energy tend to locate towards the side of the ribbon with a lower (higher) potential as the field term increases. New channels within the W-region (yellow and green in panels (h, i)) can have a mixture of valence and conduction band character, and can reside mostly on the opposite side of the ribbon to the standard sub-band channels. This gives rise to the channel segregation features discussed in the main manuscript. We note that the same four-fold degeneracy of bulk channels can occur for a higher doping level, highlighted by the valence band energy furthest from $E = 0$ (triangles in panel c). However, we note that all of the channels associated with band crossings at this Fermi energy (panel j) reside on the lower edge of the ribbon, and will not give rise to multiple sets of segregated bulk transmission channels.

Density of states and current distributions. The local current information from the c-AFM distribution in Fig 3b of the main manuscript can be compared with simulations of the local current

density and density of states (LDOS) across the ribbon. We consider an infinite nanoribbon for these simulations, with semi-infinite extensions of the device to either side acting as leads. Local bond currents between sites j and i, due to current injection from the left lead, are given by [5]

$$J_{ij} = H_{ji} \, \mathrm{Im} \, [G^R \, \Gamma_L \, G^A]_{ij} \tag{1}$$

and a local current density is calculated by averaging this quantity over a small number of sites to remove short-ranged sublattice-dependent fluctuations far beyond the resolution of the c-AFM measurement. The LDOS at site i is calculated from

$$\rho_i = -\frac{1}{\pi} \mathrm{Im} \, G_{ii} \, . \tag{2}$$

A cross section of each of these quantities across the ribbon width is shown in Supplementary Figure 6b (note the log scale for the LDOS). We note that the qualitative features of the experimental c-AFM sweep, namely a large current flow at the lower edge, and smaller, oscillating currents across the rest of the ribbon, are consistent with these results.

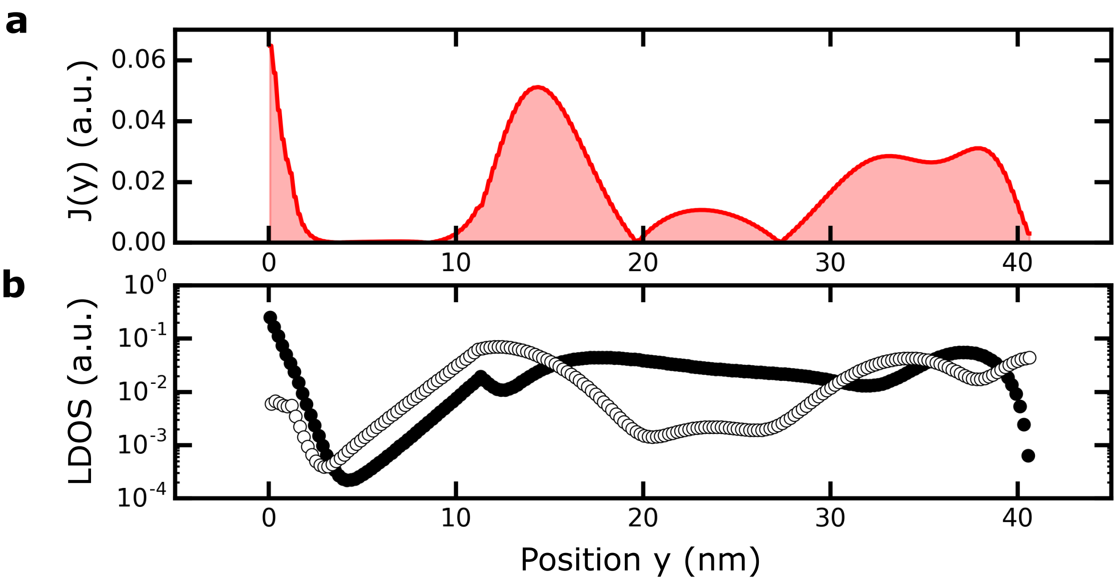

Supplementary Figure 6 | Tight-binding simulations of the local current density J and local density of states (LDOS) as a function of position across the ribbon. The parameters are the same as in the main text. Filled and hollow symbols for the LDOS plot correspond to sites from the two different sublattices.

References

1. Baringhaus, J. et al. Exceptional ballistic transport in epitaxial graphene nanoribbons, *Nature* **506**, 349 (2014).

2. Baringhaus, J. et al. Growth and characterization of sidewall graphene nanoribbons, *Appl. Phys. Lett.* **106**, 043109 (2015).

3. Carpick RW, Salmeron M, Scratching the Surface:âĂĽ Fundamental Investigations of Tribology with Atomic Force Microscopy, *Chem. Rev.* **97** 1163 (1997).

4. Berger, C. et al. Electronic Confinement and Coherence in Patterned Epitaxial Graphene, *Science* **312** 1191-1193 (2006).

5. Lewenkopf, C. H. et al. The recursive Green's function method for graphene, *J. Comput. Electron.* **12**, 203 (2013).
